# Editorial: Pathogenic potassium channel variants in neurological disorders: from functional analysis to personalized pharmacological approaches

**DOI:** 10.3389/fncel.2025.1614320

**Published:** 2025-07-30

**Authors:** Ilenio Servettini, Luca Guglielmi, Luigi Sforna, Luigi Catacuzzeno

**Affiliations:** ^1^Department of Medicine and Health Science “V. Tiberio, ” University of Molise, Campobasso, Italy; ^2^MRC Laboratory of Molecular Biology (LMB), Cambridge, United Kingdom; ^3^Department of Chemistry, Biology and Biotechnology, University of Perugia, Perugia, Italy

**Keywords:** neurological disorder, potassium channel, pathogenic variant, precision medicine, correlation genotype-phenotype

The well-established link between specific K^+^ channel mutations and neurological disorders like epilepsy, ataxia, intellectual disability, neurocognitive delay and autism spectrum disorders has been extensively studied (Sicca et al., 2016; D'Adamo et al., 2020; Guglielmi et al., 2015; Ambrosini et al., 2014; Allen et al., 2020; Cheng et al., 2021). Initial discoveries focused on a limited number of mutations, enabling detailed functional characterization in terms of the changes in the biophysical properties of the channel as well as their consequence on physio-pathological processes. However, the advent of advanced genetic sequencing and increased awareness of K^+^ channel relevance in neuronal physiology have dramatically expanded the list of pathogenic variants. These studies have highlighted how the phenotype associated to the neurological disease is often variable and likely dependent on the nature of the genetic mutation and the functional consequences produced on K^+^ channel expression and activity (Soldovieri et al., 2023; Wei et al., 2022; Cioclu et al., 2023; Bar et al., 2020). Thus, understanding the functional effects of novel disease-linked variants is key to better stratify patients, with the final aim of providing personalized therapies. Furthermore, an in-depth characterization of K^+^ channel disfunction in disease would ease drug repurposing, which has already yielded promising, albeit limited, clinical results (Hedrich et al., 2021; Ambrosino et al., 2023). Despite these advances, the pace of variant discovery has outpaced our ability to functionally characterize them, creating a widening knowledge gap. With this Research Topic, we aim to help close this gap by highlighting eight key studies providing significant new insights into the consequences of K^+^ channels mutations in neurological disorders.

Mosca et al. identified a gain-of-function (GoF) S937G variant in the *KCNT1* gene (encoding the K_Na_1.1 channel) in a girl with drug-resistant focal seizures, developmental delay, and behavioral disorders. Functional analysis using patch-clamp on transfected CHO cells confirmed the GoF phenotype. *In vitro* electrophysiology demonstrated that fluoxetine significantly reduced the aberrant current. Subsequently, fluoxetine administration in the patient led to sustained EEG improvement and seizure cessation, alongside behavioral and cognitive benefits, suggesting its potential as a precision therapy for KCNT1-related GoF epilepsies.

Nissenkorn et al. explored donepezil, an acetylcholinesterase inhibitor, as a potential therapy for developmental encephalopathy and autism caused by GoF mutations in KCNQ2/3 genes (Kv7 channels). *In vitro* studies on mouse hippocampal neurons showed donepezil reduced M-current density and increased firing frequency. A 12-month trial in four children with KCNQ2/3 GoF variants demonstrated improvements in cognitive and autistic features, suggesting donepezil repurposing as a novel treatment.

Manville, Block, et al. characterized a novel KCNB1 variant (p.S114R) in the Kv2.1 channel N-terminal region, identified in siblings with neurodevelopmental disorders. Two-electrode voltage clamp in Xenopus oocytes revealed slowed channel activation, deactivation, and inactivation, resulting in increased net current. Detailed clinical phenotyping provided strong genotype-phenotype correlation.

Manville, Illeck, et al. functionally analyzed two KCNB1 variants (P385L and P385T) in the extracellular loop preceding Kv2.1 S6. Two-electrode voltage clamp in Xenopus oocytes showed both variants caused a near-complete loss of function, both when expressed alone and when co-expressed with wild-type Kv2.1. Detailed clinical descriptions strengthened genotype-phenotype correlations.

Bernhard et al. investigated a novel KCNC3 variant (p.E675K) in a patient with atypical spinocerebellar ataxia type 13. Electrophysiology in Xenopus oocytes indicated a loss-of-function characterized by reduced current amplitude and increased cumulative inactivation, without a dominant-negative effect typical of other SCA13 variants. *In vitro* drug testing yielded no positive modulators. Detailed clinical data provided insights into this rare presentation.

Filareto et al. focused on personalized pharmacological strategies for drug-resistant pediatric epilepsy due to K^+^ channel variants. Functional analysis of missense variants in Kv7.2, Kv7.3, Kv3.1, and K_Na_1.1 channels revealed loss-of-function for Kv7 variants and gain-of-function for Kv3.1/K_Na_1.1 variants. Gabapentin reversed Kv7 loss-of-function *in vitro*, while fluoxetine counteracted Kv3.1/K_Na_1.1 gain-of-function. Subsequent treatment of patients based on these findings showed significant clinical improvements.

Faulkner et al. highlighted the central role of Kv3 voltage-gated K^+^ channels in various neurological and psychiatric disorders. Given their functional properties and neuronal expression, Kv3 channels are proposed as promising drug targets, currently lacking specific clinical modulators.

Rajkumar et al. proposed K^+^ channels as potential therapeutic targets in non-Mendelian psychiatric syndromes, particularly Post-traumatic Stress Disorder (PTSD). Evidence from animal models suggests the involvement of multiple K^+^ channel families in PTSD-like phenomena, suggesting that developing effective and safe channel modulators could significantly advance PTSD management.

## Conclusion

Although there is still much work to be done, we can consider the articles published in this Research Topic a step toward a translational approach based on the functional characterization of K^+^ channel pathogenic variants, aiming at both a better genotype-phenotype correlation and a targeted therapeutic approach. In addition, the relevance of identifying specific modulators of K^+^ channels is highlighted, both for Mendelian and non-Mendelian pathologies.
